# Generating Electricity during Walking with a Lower Limb-Driven Energy Harvester: Targeting a Minimum User Effort

**DOI:** 10.1371/journal.pone.0127635

**Published:** 2015-06-03

**Authors:** Michael Shepertycky, Qingguo Li

**Affiliations:** Bio-Mechatronics and Robotics Laboratory, Department of Mechanical and Materials Engineering, Queen’s University, Kingston, Ontario, Canada; University of Alberta, CANADA

## Abstract

**Background:**

Much research in the field of energy harvesting has sought to develop devices capable of generating electricity during daily activities with minimum user effort. No previous study has considered the metabolic cost of carrying the harvester when determining the energetic effects it has on the user. When considering device carrying costs, no energy harvester to date has demonstrated the ability to generate a substantial amount of electricity (> 5W) while maintaining a user effort at the same level or lower than conventional power generation methods (e.g. hand crank generator).

**Methodology/Principal Findings:**

We developed a lower limb-driven energy harvester that is able to generate approximately 9W of electricity. To quantify the performance of the harvester, we introduced a new performance measure, total cost of harvesting (TCOH), which evaluates a harvester’s overall efficiency in generating electricity including the device carrying cost. The new harvester captured the motion from both lower limbs and operated in the generative braking mode to assist the knee flexor muscles in slowing the lower limbs. From a testing on 10 participants under different walking conditions, the harvester achieved an average TCOH of 6.1, which is comparable to the estimated TCOH for a conventional power generation method of 6.2. When generating 5.2W of electricity, the TCOH of the lower limb-driven energy harvester (4.0) is lower than that of conventional power generation methods.

**Conclusions/Significance:**

These results demonstrated that the lower limb-driven energy harvester is an energetically effective option for generating electricity during daily activities.

## Introduction

Battery capacity has a critical impact on the performance and operation time of portable devices, such as GPS and cell phones. To achieve a longer duration of operation, energy harvesting technology was evaluated as an alternative to carrying extra batteries [1–4]. An ideal energy harvester should generate a substantial amount of electricity with a minimal increase in user effort. In addition, it should operate in concert with the user during daily activities such as walking and jogging, without disturbing his/her natural movement. These requirements inspire studies of biomechanics and energetics of energy harvesting through human experimentation.

There are currently two energy harvesters that are capable of generating electricity at a 5W level. The first is a suspended backpack [5] that captures the vertical oscillations of a 38kg load and generates 7.4W of electricity during fast walking. The same device generates 3.7 W while walking with a 29kg load at 1.5 m/s. The second device is a knee-mounted device [2] that assists the knee deceleration during the swing phase and generates 4.8W of electricity. To quantify the energetic consequence of energy harvesting on the user, cost of harvesting (COH) was first introduced in [2], where COH is defined as the amount of metabolic power (W) required to generate 1W of electricity. The suspended backpack’s COH was calculated to be 4.8. By assisting the knee flexor muscles during the deceleration phase, the knee-mounted harvester achieved a COH of 0.7, which is very efficient in generating electricity. Since the metabolic power increase in COH calculation is defined as the difference between walking while generating electricity and walking while carrying the device without generating electricity, COH only measures the metabolic efficiency for power generation. Without considering device carrying cost, solely comparing harvesters’ COH can be misleading, because COH does not reflect the overall energetic consequence of using an energy harvester. To fully quantify the energetic effect of an energy harvester on the user, the device carrying cost should also be considered. Here, we propose a new measure, total cost of harvesting (TCOH), which is the ratio between the metabolic power increase from normal walking (without carrying the harvester) and the amount of electrical power produced.

Despite the knee harvester being efficient in generating electricity (COH = 0.7), the device’s TCOH is 13.6, which is 19 times larger than its COH. Experiments showed that walking with the knee harvester increased the energy expenditure by 20% when compared to walking without the device [2]. Consequently, the carrying cost of the distally located device diminished the energetic benefit achieved through generative braking. Similarly, when considering the carrying cost of 29kg load, the suspended backpack has a higher overall harvesting cost with a TCOH of 30.7. However, if the user (e.g. solider) is already obliged to walk with a 29kg load, this weighted carrying scenario becomes his/her normal walking condition. The suspended backpack’s estimated TCOH drops to 7.5. It is clear that TCOH could serve as a better metric for comparing different energy harvesting devices. In addition, TCOH could be used to quantify whether an energy harvester is an energetically economical option in comparison with conventional power generation methods, such as a hand crank generator.

To achieve a low TCOH, a harvester needs to simultaneously have a low COH and a low carrying cost. A low COH requires the device to operate in generative braking mode [2], where most of the electricity is generated from negative muscle work. To reduce the carrying cost, the device should be light weight and located close to the user’s center of mass (COM), because the metabolic cost of carrying a given mass increases as it moves distally from the user’s COM to the lower limbs [6, 7]. In addition, the energy harvester should not alter the user’s preferred walking mechanics, because any deviation from the user’s natural gait (e.g. step length, frequency [8], width [9]) increases the metabolic cost of walking. By reducing device carrying cost and generating electrical power through generative braking, energy harvesting would be more efficient than conventional power generation methods. We tested this hypothesis in controlled human experiments with a lower limb-driven energy harvester.

## Materials and Methods

### Ethics Statement

The experiment was approved by the General Research Ethics Board of Queen’s University (GREB Romeo #:6006569) and written informed consent was obtained from all subjects.

### The Device

**Fig 1:**
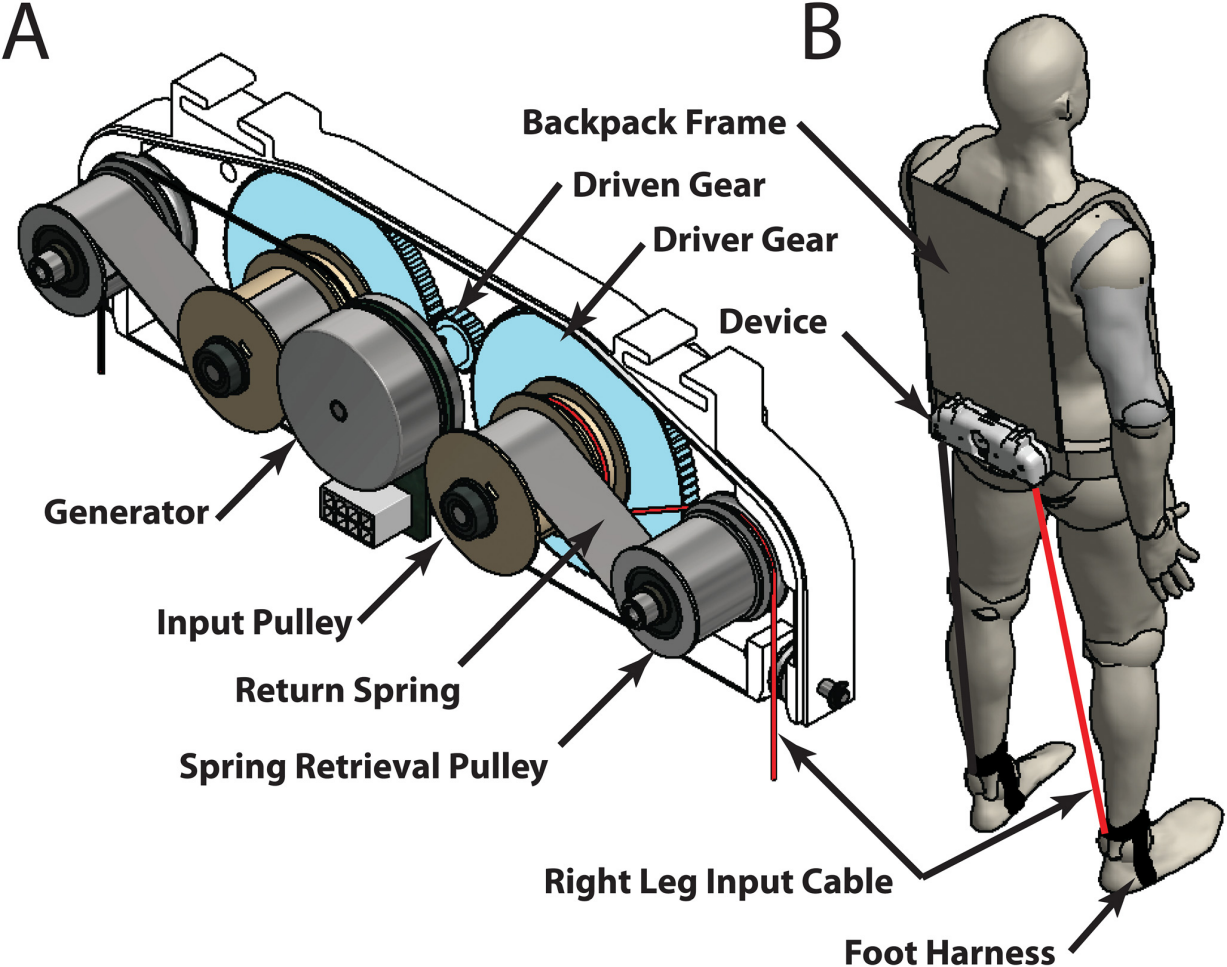
Lower-limb driven energy harvester. **(A)** Schematic of device components. **(B)** Schematic view of the device worn by the user. The lower limbs pull the cables during the swing phase of a walking cycle and the out-of-phase motion of the two limbs makes the integration of the two limb motion into a single generation unit possible.

We developed a lower limb-driven energy harvester that captures the motions of the user’s lower limbs during the swing phase of gait cycle with a single power generation unit (Fig 1). We aimed to achieve a low TCOH by: A) decreasing device carrying cost by minimizing its weight and locating it near the user’s COM, and B) increasing the amount of electrical power produced through harnessing negative muscle work of both legs.

**Fig 2:**
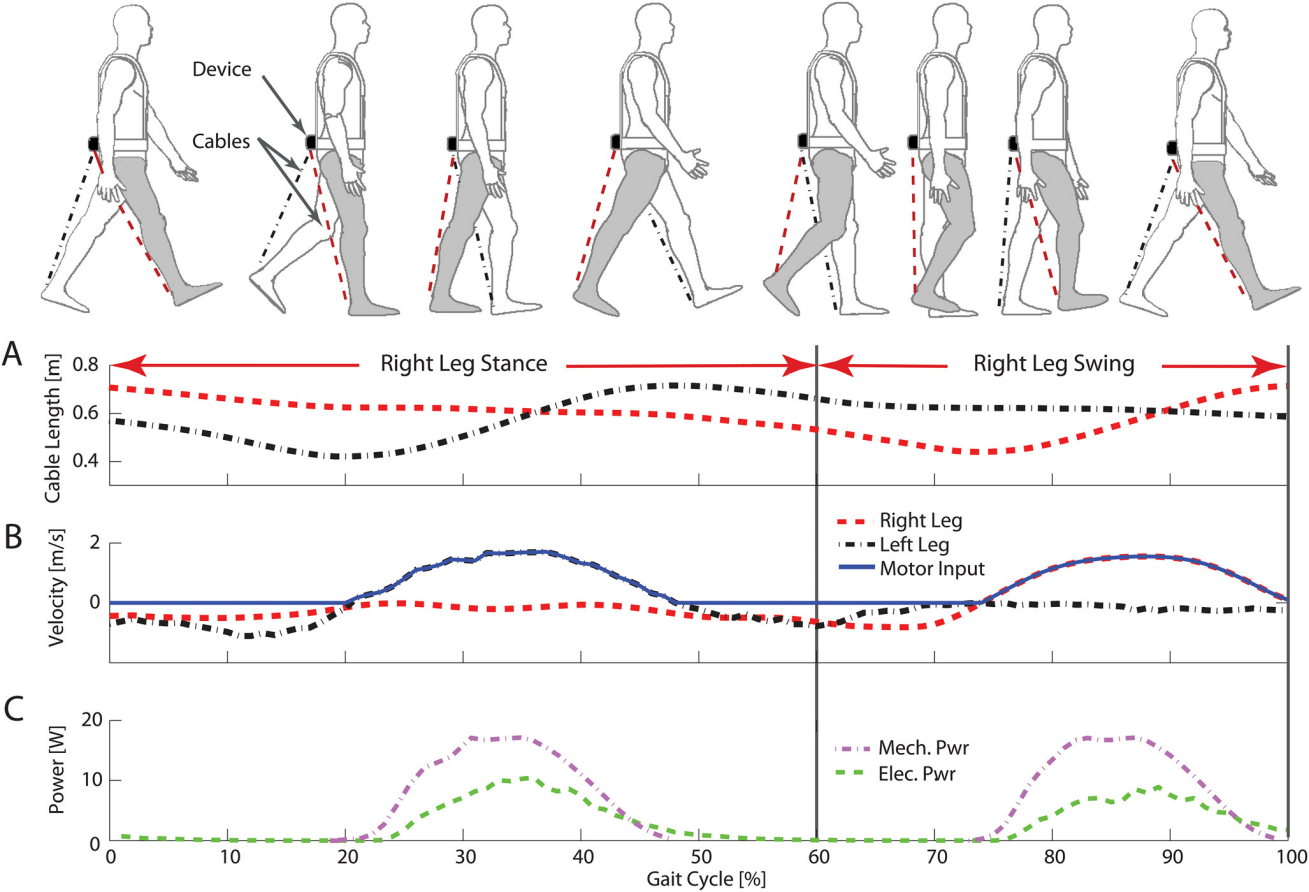
Timing of power generation during walking. **(A)** The right (red) and left (black) leg cable length [m] in a stride cycle **(B)** The right (red) and left (black) leg cable velocities [m/s] and the velocity input into the harvester (blue), during a gait cycle. The harvester combines the positive velocity of the two cables because the cables only pull the power generation unit during their lengthening periods. **(C)** Mechanical power (purple) exerted by the user and the electrical power (purple) produced by the lower-limb driven energy harvester.

This harvester captures the linear displacement between the user’s hip and ankle during walking through two input cables and generates electricity (Fig 2). For a given leg, the cable length increases during the swing phase and starts to decrease after heel-strike. In the late swing phase, the knee flexor muscles perform negative work to decelerate the knee motion. The resistance applied by the harvester assisted the knee deceleration, which is similar to the generative braking mode used in the knee harvester [2]. Because the cable extension periods of the two limbs are out of phase, the harvester was able to capture the motion of two limbs into a single power generation unit. This design significantly reduced the device complexity and weight. Because the mass location strongly affects the metabolic cost of weight carrying [6, 7], we mounted the power generation unit near the user’s COM at the bottom of a backpack frame. One end of each cable was attached to the user’s lower shank through a custom-made foot harness and a quick release clip, while the other end was attached to its respective input pulley (one pulley for each cable). The quick release clips allowed the cables to be easily detached and retracted into the device in cases such as sitting. The cables were attached to the user’s lower shanks to prevent the device from applying a moment on the ankle joint. Custom-designed input pulleys converted the linear cable motion into rotation. A unidirectional roller clutch was inserted between the driving shaft and the driver gear. The roller clutch engaged the gear with the shaft in one direction (cable extension) and allowed free motion in the opposite direction (cable retraction). This facilitated for a single leg to drive the system during it’s respective swing phase, which decoupled the motions between two limbs. A constant force spring (Load: 0.1kgf, MiSUMi, USA) mechanism was designed to retrieve the cables prior to the start of the next cycle (Fig 1.A). The spring mechanisms provided under 1N of tension on the cables and therefore provided a negligible resistance to the user during static conditions such as the stance phase of walking. The input rotation was amplified by a gear train (1:5 gear ratio), prior to engaging an AC generator (EC-4pole 200W, Maxon Motor, Switzerland). The electrical power generated from the generator was rectified using a three-phase, full bridge rectifier and the electrical power was currently dissipated with high-power resistors. The total system weight is 2.66kg (device weight: 0.95kg, backpack weight: 1.19kg, rectifying circuit: 0.39kg, shoe harnesses: 0.13kg).

### Human Experimentation Protocol

Human walking experiments were conducted at the Human Performance Laboratory (Hotel Dieu hospital, Kingston, ON) to evaluate the performance of the harvester and determine the effects of the harvester on the user’s kinematics, kinetics, and energetics. Ten young, healthy, adult males were recruited to participate in this study (24±3years old, 1.78±0.08m, 75.6±10.4 kg). None of the participants reported any known or apparent injuries that would affect their gait, and all of them reported to live a healthy lifestyle, exercising at least three times per week. Each participant performed eight randomized treadmill-walking activities. These activities were: 1) Normal walking, where the user walked without wearing the energy harvester; 2) Weight-only walking, where the user walked while wearing the harvester without the cables attached; 3) Mechanical engagement, where the user walked while wearing the energy harvester with the cables connected, but with electrical power generation turned off by leaving the circuit open; and 4–8) Electrical engagement, where the user walked with the energy harvester while generating electricity at five electrical resistances (19Ω, 11 Ω, 6 Ω, 4Ω, and 2.5Ω). Lower electrical resistances were associated with greater mechanical load felt by the user. Each activity lasted 10 minutes and the activities were separated by a 3-minute rest period. All walking trials were conducted on a split-belt AMTI Force-Sensing Tandem Treadmill (AMTI Inc., MA).

Each participant had two acclimation periods prior to data collection. The first acclimation period was conducted the day prior to testing. The participant walked on a single-belt treadmill with the device harvesting electricity (6 Ω resistance) for 10 minutes. This second acclimation period was conducted on the testing day, prior to the treadmill-walking activities, for five minutes. The resistance used for this acclimation period was randomly selected. This specific resistance was removed from the electrical engagement trials to avoid any bias caused by learning effect. After the acclimation, on the testing day, the participant’s resting metabolic power during 10-minute quiet standing was measured.

### Device Performance

The electrical power production and the device efficiency were determined for electrical engagement trials. The electrical power was calculated as the product of the voltage and current, measured across the electrical resistances at a sampling rate of 1000Hz. The mechanical power was calculated as the product of the cable force and cable velocity. The cable force was measured using a load cell (LC201, Omega, USA) that was inserted between the shoe harness and input cable of the right leg. The cable force was sampled at a frequency of 1000Hz with a data acquisition card (USB-2533, Measurement Computing, MA). The cable velocity was determined by differentiating the relative cable length. The cable length was measured as the distance between reflective markers placed on the cable attachment point on the foot harnesses and on the cable insertion point on the harvester. The device efficiency was calculated as the ratio between electrical power and mechanical power input.

### Metabolic Power and Total Cost of Harvesting

The energetic consequence of using the energy harvester was determined from the rate of oxygen consumption and carbon dioxide production, which were measured using an open respirometry (K4b2, COSMED, Italy). Metabolic power was calculated for each trial using the standard equation from [10]. Metabolic data from the third quarter (minutes 5 to 7.5) of each trial was analyzed to allow the participant to reach steady state and to prevent end-effects. Each participant was cleanly shaven and asked to refrain from eating four hours prior to testing. For each walking trial, the net metabolic change was calculated by subtracting the resting metabolic power from the metabolic power calculated during that walking trial.

To quantify the user effort in generating electrical power, COH and TCOH were calculated for each electrical engagement trial. COH, originally proposed in [2], represents the additional metabolic power required to generate 1W of electrical power in comparison with weighted walking,

$$\begin{array}{c} \text{COH}=\frac{\;\text{metabolic}\;\text{power}\;\text{of}\;\text{elec.}\;\text{engagement-metabolic}\;\text{power}\;\text{of}\;\text{weighted}\;\text{walking}}{\text{electrical}\;\text{power}} \end{array}$$ (1)

The TCOH relates the additional metabolic power required to generate 1W of electrical power in comparison with normal walking,

$$\begin{array}{c} \text{TCOH}=\frac{\;\text{metabolic}\;\text{power}\;\text{of}\;\text{elec.}\;\text{engagement-metabolic}\;\text{power}\;\text{of}\;\text{normal}\;\text{walking}}{\text{electrical}\;\text{power}} \end{array}$$ (2)

Comparing the two measures, TCOH provided a better metric in quantifying the user’s overall effort in harvesting electricity. It considered both the cost of electrical power generation and the device carrying cost, which is an unavoidable contributor to the user’s metabolic cost when using an energy harvester.

### Joint Kinematics and Kinetics

During each walking trial, the sagittal joint kinematics of the right hip, knee, and ankle were found using a seven-camera motion capture system (Oqus, Qualisys, Sweden). A modified Cleveland Clinic lower limb marker set was used to track the trunk and lower limb motion. The modified marker set assumed that the trunk and pelvis combined into a single rigid body so that the pelvis could be tracked using markers placed on the left and right acromion, sternal notch, and the C7 vertebrae. This modification was necessary because the hip-belt of the backpack covered the standard pelvis tracking markers (right and left anterior superior iliac spine and posterior superior iliac spine; PSIS).

The ground reaction force was measured using an AMTI Force-Sensing Tandem Treadmill (AMTI Inc., MA) at a sample frequency of 1000Hz. The ground reaction forces was then filtered using a second-order Butterworth low-pass filter with a cutoff frequency of 10Hz. The segment anthropomorphic data were estimated and scaled using standard regression equations [11] based on the participant’s weight and segment length measurements.

**Fig 3:**
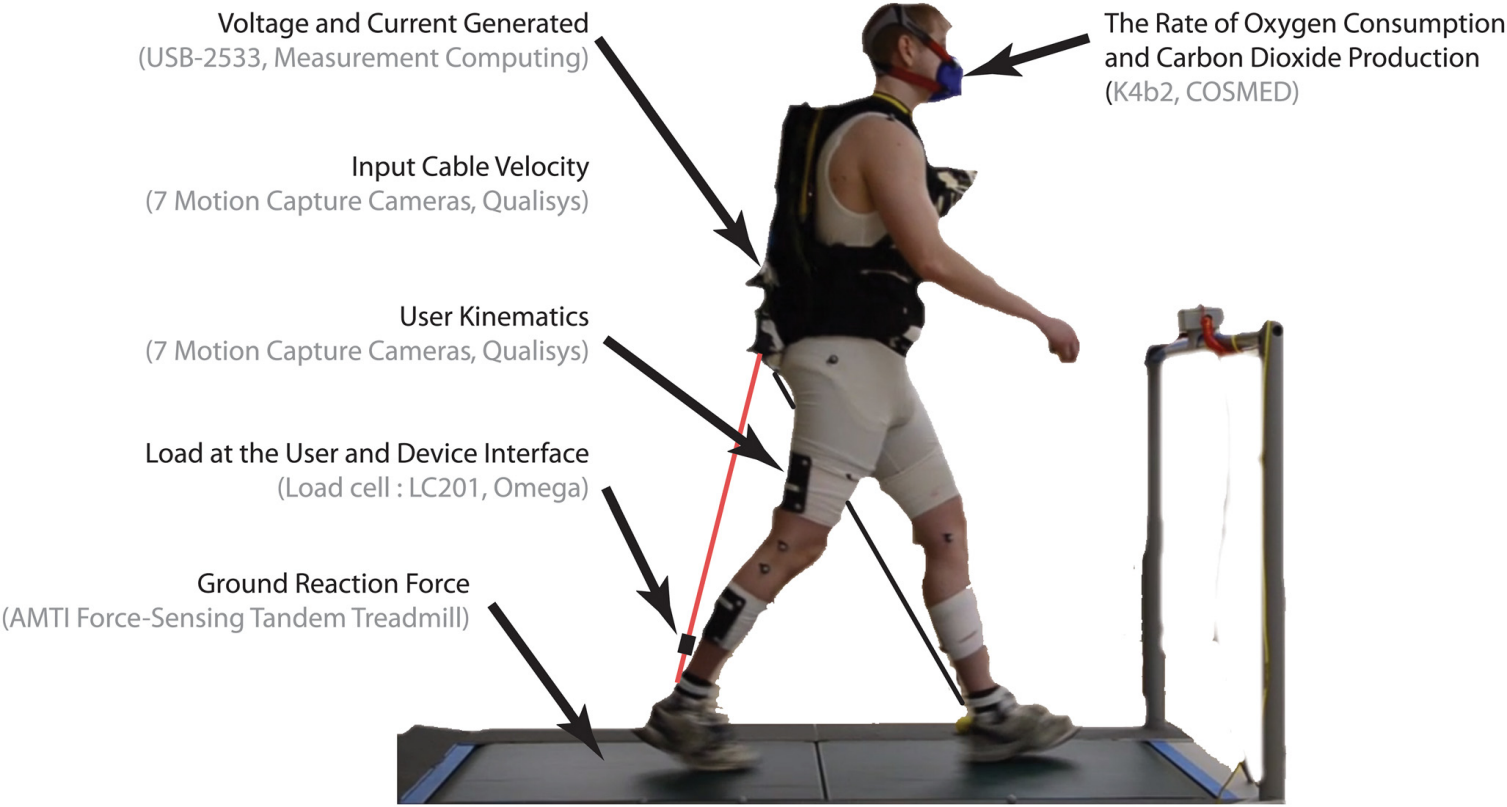
Human experimentation setup. The user’s kinematics, kinetics, and energetics were measured along with device’s input mechanical power and electrical power output.

The first eight consecutive gait cycles after 5 minutes of walking were used to calculate joint angle, moment, and power for each joint. The analysis was conducted using a custom MATLAB script (Mathworks, MA). Cycles were excluded if both feet were simultaneously placed on a single force plate. The participants’ mean joint angles, moments, and powers over these eight gait cycles were calculated. These net joint moments and powers were normalized to body weight. The grand mean of each walking trial was calculated from the individual mean of each participant. The start of a gait cycle was considered to occur when the right heel made contact with the front force plate. With an activation threshold of the force measurement at 5N, the step length and step width were calculated as the fore-aft and medial-lateral distance between the left and right heel markers at the heel-strike events. The range of motion of the participant’s right knee was found for each of the eight consecutive gait cycles, by calculating the difference, in degrees, of the maximum and minimum knee angle in that gait cycle. Similarly, the participant’s COM displacement was calculated as the difference between the maximum and minimum vertical positions of a virtual marker located between the digitized PSIS [12]. These virtual marker locations were found using a static calibration and tracked using the upper body markers listed above. The human experimental setup and measurement equipment are listed in Fig 3.

### Statistical Analysis

To quantify the effect of the lower limb-driven energy harvester on the user’s walking kinematics and kinetics, statistical analysis was performed to compare five dependent variables: step-length, step-width, maximum knee torque, knee range of motion, and COM deflection. Statistical comparisons were performed using a repeated measures ANOVA for each dependent variable (within subject variable: walking trial; 8 levels) (*α* = 0.05). If a significant difference was found, post hoc comparisons were performed using Sidak-Holm step-down paired t-tests.

## Results and Discussion

### Device Performance

**Fig 4:**
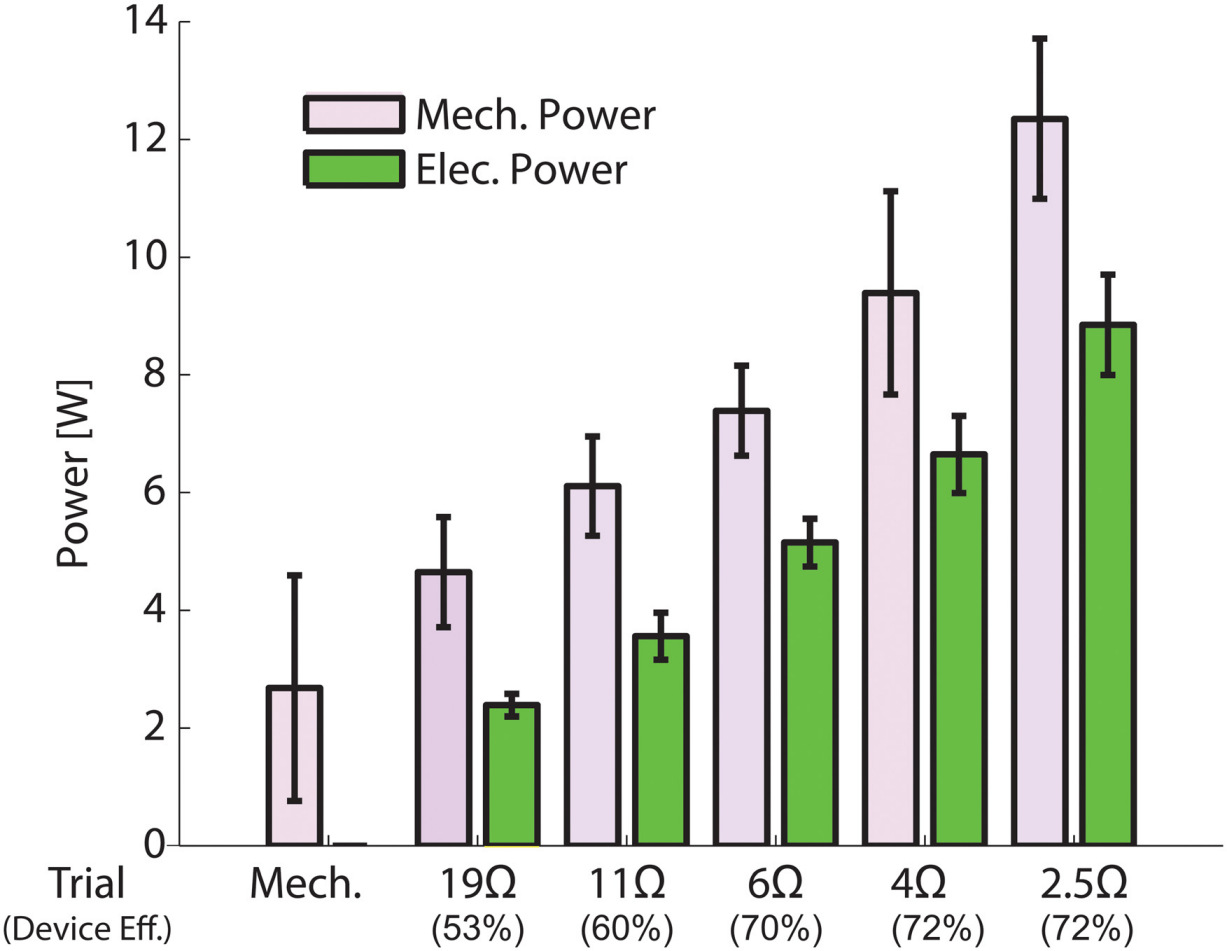
Mechanical and electrical power of the lower-limb driven energy harvester. The mechanical power (purple) was calculated as the product of the input cable velocity and the cable force; the electrical power (green) was calculated as the product of the measured output voltage and current of the electrical resistor. The efficiency was calculated as the ratio between the electrical power and mechanical power.

The electrical power produced by the lower limb-driven energy harvester increased from 2.4±0.2W for an electrical resistance of 19Ω to 8.9±0.9W for an electrical resistance of 2.5Ω (Fig 4). The associated mechanical power were 4.6±0.9 to 12.3±1.4. The device efficiency improved from 53% to 72% as the electrical resistance decreased from 19Ω to 4Ω and reached a plateau of 72% at resistances of 4Ω and 2.5Ω (Fig 4).

The total mechanical power input was made up of two components: power for driving the mechanical components of the harvester and power for generating electricity. The mechanical engagement condition indicated that on average 2.7±1.9W of mechanical power was required to run the device components. The initial improvement in efficiency was due to the increase in the amount of electricity generated, which made the mechanical power for driving the device components a smaller portion of the total input mechanical power. With further reduction of electrical resistance, the output current increased and more electrical energy was dissipated in the internal winding resistance of the motor (0.386Ω for EC-4 pole 200W motor). This led to the later plateau observed in device efficiency.

### Metabolic Power and Total Cost of Harvesting

Our experiments found that the weighted walking condition had a 26±21*W* metabolic increase compared to the normal walking condition. The metabolic cost for the mechanical engagement and electrical resistance trials (19Ω, 11Ω, 6Ω, 4Ω, and 2.5Ω), relative to weighted walking, were −8±5, −5±14, 2±11, −4±23, 7±20, and 30±19, respectively.

**Fig 5:**
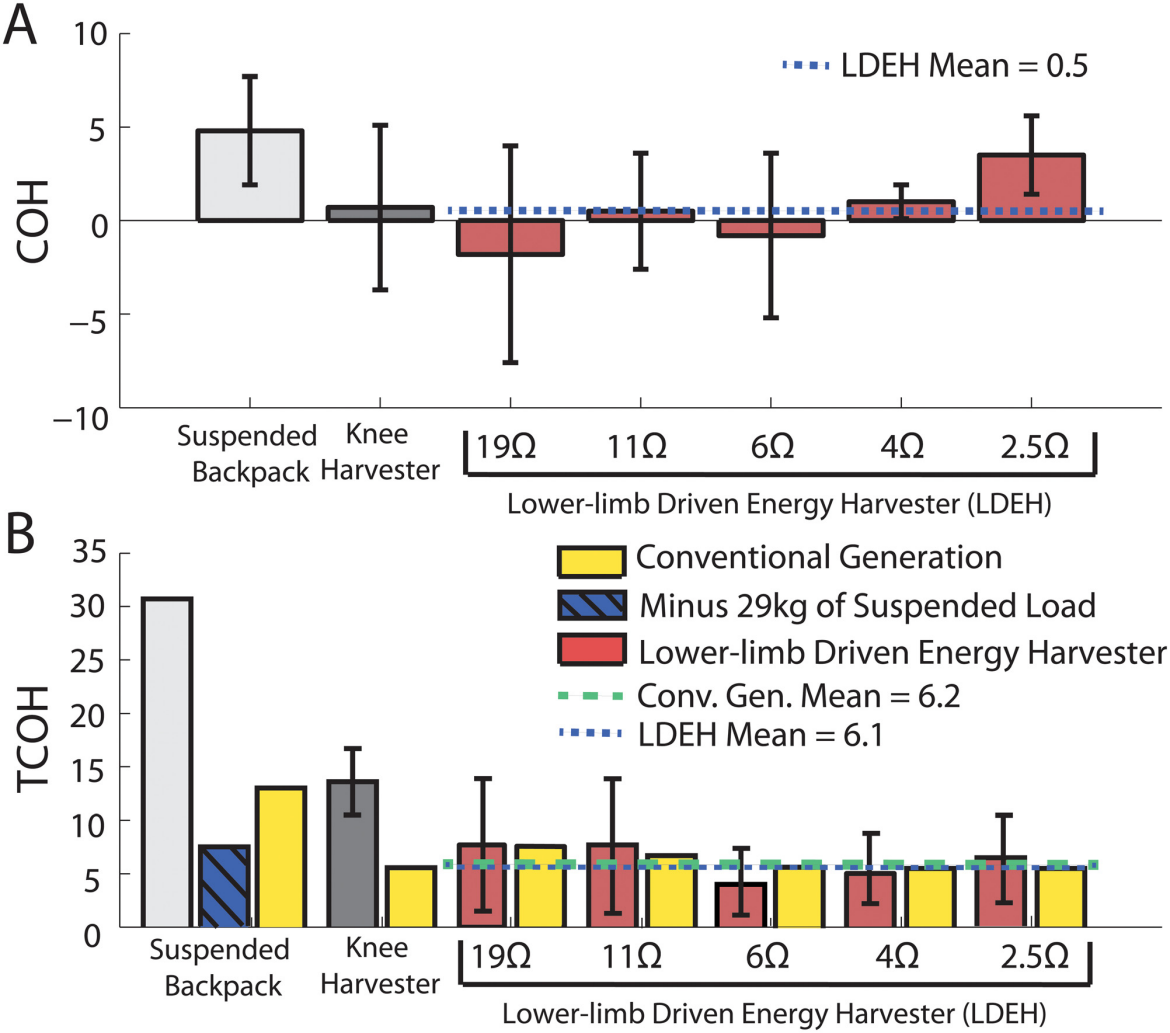
Cost of harvesting and total cost of harvest. **(A)** The mean cost of harvesting (COH) for the lower-limb driven energy harvester (Red) (for electrical resistances of 19Ω, 11Ω, 6Ω, 4Ω, and 2.5Ω) in comparison with the suspended load backpack (light gray) [5], and knee harvester (dark gray)[2].The mean COH over all five electrical resistance conditions for the lower-limb driven energy harvester (blue dotted line). **(B)** The mean total cost of harvesting (TCOH) for the lower-limb driven energy harvester(red) under different electrical resistances (electrical resistances of 19Ω, 11Ω, 6Ω, 4Ω, and 2.5Ω), suspended load backpack (light gray when compared with normal walking condition, hatched blue when compared with weighted walking condition) [5], and knee harvester (dark gray)[2], in comparison with each device’s estimated COH for convention generation (yellow).

When relating the metabolic cost to the amount of electrical power generated, the COH of the lower limb-driven energy harvester ranged from −1.8±5.8 to 3.5±2.1 for electrical resistances of 19Ω and 2.5Ω, respectively (Fig 5.A). The mean COH for all five electrical engagement conditions was 0.5±2.0, indicating that the lower limb-driven energy harvester used approximately 0.5W of metabolic power to produce 1W of electricity. This number is slightly smaller than the COH of the knee-mounted device (0.7±4.4) [2]. Under three intermediate resistance conditions (19Ω, 11Ω, and 6Ω), the device achieved a near zero or negative COH, which implies that most of the electrical power was generated through assisting negative muscle work.

When considering device carrying cost, the TCOH ranged from 7.7±6.2 to 4.0±3.6, for the electrical resistances used in the experiment (Fig 5.B). The mean TCOH for all five electrical engagement trials was 6.1±1.6, which is less than half of the TCOH of the knee-mounted device (13.6±3.1)[2]. Because the suspended backpack was designed for harvesting electricity with a substantial load being carried, it is difficult to directly compare TCOH with the harvester in this study. The TCOH of suspended backpack can be estimated in two ways depending on the condition to which it compares with. When comparing with the normal walking, the TCOH of the suspended backpack is calculated as 30.7 based on data provided in [5]. When considering walking with the 29kg load as the normal walking condition, the TCOH can only be estimated as the exact weight of the device is not available. To get an estimate, we assume the energy harvesting components of suspended backpack weighs the same as the lower limb-driven harvester (2.66kg). With a device location factor of 3.3W/kg for the waist [7], the weight could have caused a metabolic increase of 8.8W. Adding 19W of power generation cost (producing 3.71W of electricity) [5], the total metabolic increase is 27.8W. The estimated TCOH would be 7.5, which is slightly higher than the harvester in this study.

To further compare the lower limb-driven harvester with a conventional power generation method (e.g. hand crank), the COH for a conventional power generation was estimated using the following equation according to [2],

$$\begin{array}{c} \text{COH}\;\text{for}\;\text{conventional}\;\text{generation}=\frac{1}{\eta_{d}\times \eta_{m}} \end{array}$$ (3)

where *η*_*d*_ is the device efficiency at the same electrical resistance and *η*_*m*_ is the muscle’s peak efficiency in performing positive work (25%)[13]. The COH for a conventional power generation method for each electrical resistance is shown in Fig 5.B, with a mean COH of 6.2±0.9. The knee harvester’s TCOH was higher than it’s respective conventional generation COH (6.4). When not considering the cost of carrying the 29kg load, the suspended backpack’s TCOH was also lower than its conventional generation (12.8). When accounting for the device carrying cost, the proposed lower limb-driven energy harvester is still a viable energy efficient option in generating electricity.

Linking the mechanical power and the metabolic power provided insights on the device assistance of positive and negative muscle work. Under the mechanical engagement condition, the device applied 2.7W of mechanical power to the user which saved the user 8W of metabolic power. Assuming that the device only assisted negative muscle work, with muscle efficiency of -120% in performing negative work [13], the maximum theoretical metabolic decrease should be only 2.3W. However, the user experienced an additional 5.3W metabolic benefit that cannot be accounted by the negative work assistance. This led us to believe that the device might also have assisted a portion of positive muscle work in the form of reducing muscle co-contraction. The observed reduction in metabolic cost, also seen in other studies [14], could also come from other unknown mechanisms.

The measured metabolic increase associated with device carrying was higher than the estimated value based on previous studies [7, 15]. This difference was likely due to limitations in our experimental setup and measurement procedure. Participants were tethered from the back with a bundle of cables (weighing approximately 1kg) to the data acquisition board for measurements. The cables could have applied a force on the participants, which would have caused a metabolic increase [16]. Based on the previous study on the carrying cost for weight at different locations, the total carrying cost of the lower limb-driven energy harvester was estimated. With 2.53kg of the device mass being carried at the waist and 0.13kg being carried at the ankle, the estimated carrying cost was about 10.3W. This was done by summing the cost associated with carrying each portion of the device on different body segments with the device location factors (14.8 and 3.3W/kg for the ankle and waist, respectively [7]). The measured device carrying cost was 15W higher than the estimated cost. This indicates that the TCOH obtained in the experiment was the upper limit and the true TCOH of the device could be even smaller.

### Joint Kinematics and Kinetics

**Fig 6:**
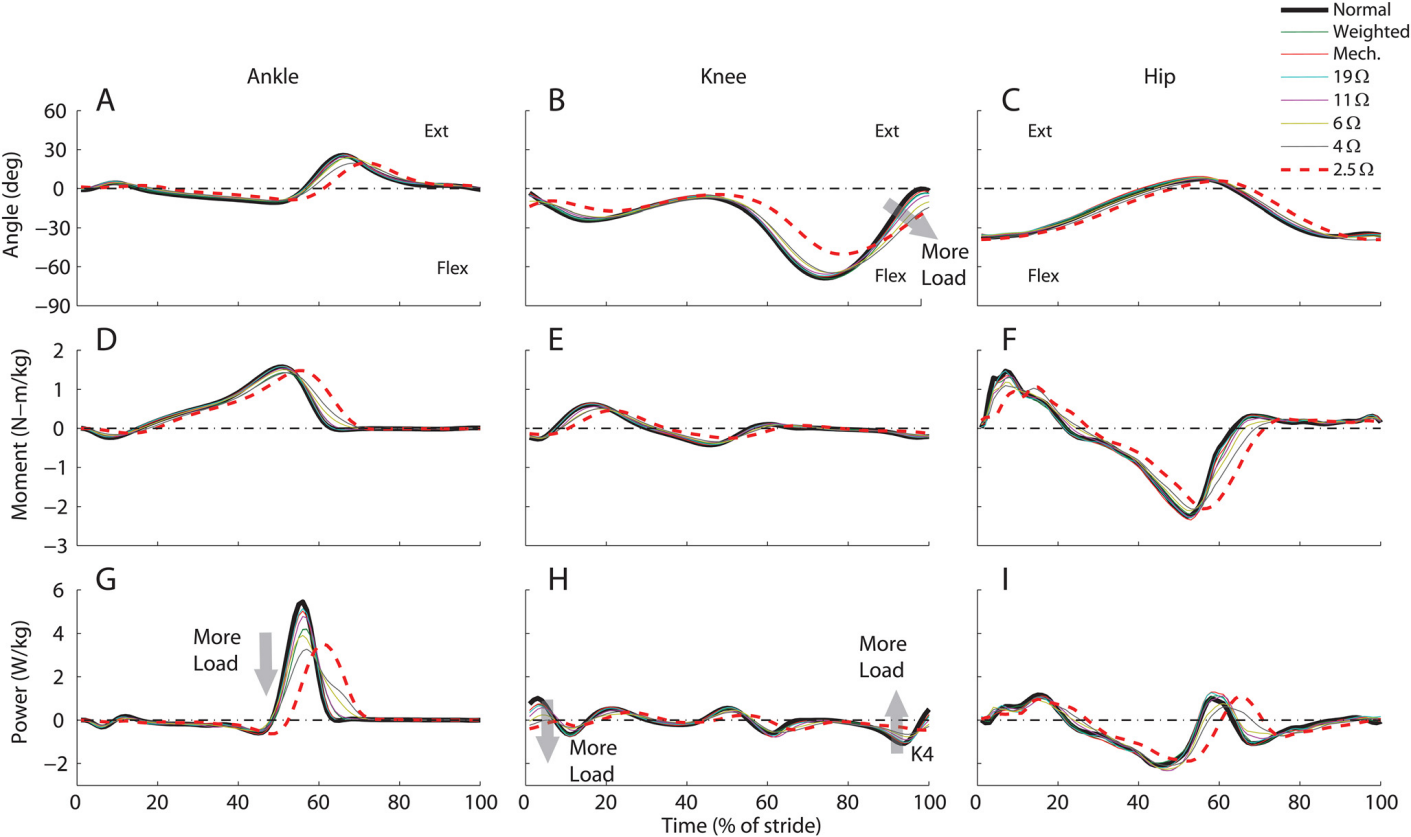
Joint kinematics and kinetics. Mean (sagittal plane) joint angles (A-C), moments (D-F), and powers (G-I), for the ankle, knee and hip of the right leg over the stride from heel-strike (0%) to heel-strke (100%), for all recorded trials (normal walking (thick black), weighted (green), mechanical engagement (solid red), and electrical engagement with resistances of 19Ω (cyan), 11Ω (purple), 6Ω (yellow), 4Ω (grey), and 2.5Ω (dashed thick red))

The kinematic and kinetic analysis of the user’s gait provided insights on the metabolic changes observed in different electrical resistance conditions. Joint kinematics (ankle, knee and hip) were similar between normal walking, weighted walking, mechanical engagement, and electrical engagement for resistances of 19Ω and 11Ω (Fig 6A–6C). When the resistance reached 6Ω, the ankle and knee joint angle started to deviate from those in the normal walking condition. For the knee joint, most of the differences occurred at the end of the swing phase of the gait cycle, where the harvester applied the braking force to the knee joint. The knee joint tended to be more flexed at the heel strike event and the range of motion of the knee became smaller. The range of motion of the knee was significantly reduced in the 2.5Ω, 4Ω, 6Ω, and 11Ω electrical resistance conditions (*P* = 0.007, *P* = 0.009, *P* = 0.010 and *P* = 0.013, respectively, Table 1). This led to the reduction of the knee joint negative power in region K4 (Fig 6H). As the electrical resistance decreased, full knee extension progressively shifted from the end of swing to the double-support phase. For the resistances of 4Ω and 2.5Ω, the knee extension was even shifted to the initial double support phase (Fig 6B) and the larger deviation from normal walking occurred at the lowest resistance condition (2.5Ω). This shift was most likely a result of the user’s response to the harvester resistance at the end of the swing phase with a smaller knee joint angle. Knee extension during the initial double support phase required the knee extensor muscles to perform extra mechanical work to raise the user’s COM, a potential source of higher metabolic cost. Previous studies have shown that the increase of flexion in the stance leg led to greater muscle force required for body weight support [17]. In addition, with the lower electrical resistance, the vertical displacement of the user’s COM was reduced, which may have also contributed to the high metabolic cost [18].

**Table 1:** Kinematic and kinetic parameters.

Trial	Step Length [m]	Step Width [m]	Knee Torque (max, min) [Nm/kg]	Knee Range of Motion [Degrees]	COM Displacement [cm]
Normal	0.72 ± 0.05	0.11 ± 0.02	0.6 ± 0.2, −0.5 ± 0.2	69 ± 5	5.3 ± 1.1
Weighted	0.68 ± 0.06	0.10 ± 0.04	0.7 ± 0.2, −0.4 ± 0.1	68 ± 5	5.4 ± 1.1
Mechanical	0.70 ± 0.04	0.11 ± 0.03	0.7 ± 0.2, −0.5 ± 0.2	67 ± 5	5.4 ± 1.1
19Ω	0.69 ± 0.06	0.10 ± 0.03	0.6 ± 0.3, −0.5 ± 0.2	65 ± 6	5.1 ± 1.3
11Ω	0.66 ± 0.06	0.12 ± 0.05	0.6 ± 0.2, −0.4 ± 0.1	63 ± 3*	5.1 ± 1.1
6Ω	0.64 ± 0.11	0.10 ± 0.03	0.6 ± 0.2, −0.5 ± 0.2	62 ± 4*	4.9 ± 1.1
4Ω	0.60 ± 0.08*	0.12 ± 0.04	0.6 ± 0.2, −0.4 ± 0.1	60 ± 6*	4.9 ± 1.1
2.5Ω	0.56 ± 0.10*	0.12 ± 0.04	0.6 ± 0.3, −0.4 ± 0.2	59 ± 5*	5.0 ± 1.3

Mean ± Standard deviation of step length [m], step width [m], knee range of motion [Degrees], max. and min. knee torque [Nm/kg] and COM displacement [cm] (over 8 consecutive steps) for all record trials.

* indicates significant difference with *P* < 0.05.

As the electrical resistance decreased, the peak ankle power during push-off decreased (Fig 6G). This was mainly due to the decrease in step length during the lower electrical resistance conditions (Table 1). It is possible that the step length/frequency might have also contributed to the high metabolic cost associated with lower electrical resistances. Studies have shown that the metabolic cost of walking increases if the step length/frequency or step width moves away from their optimal combination [9, 19, 20]. The step length during the 4Ω and 2.5Ω conditions were significantly reduced compared to the normal walking condition (P = 2×10^−5^ and P = 3×10^−4^ respectively, Table 1). Under other walking conditions, the step length/frequency did not significantly change. The device did not significantly alter the step width in any of the conditions. Investigations of powered exoskeleton-assisted walking have also demonstrated that altering gait kinematics from normal walking causes increases in metabolic cost [21], which is consistent with current study.

**Fig 7:**
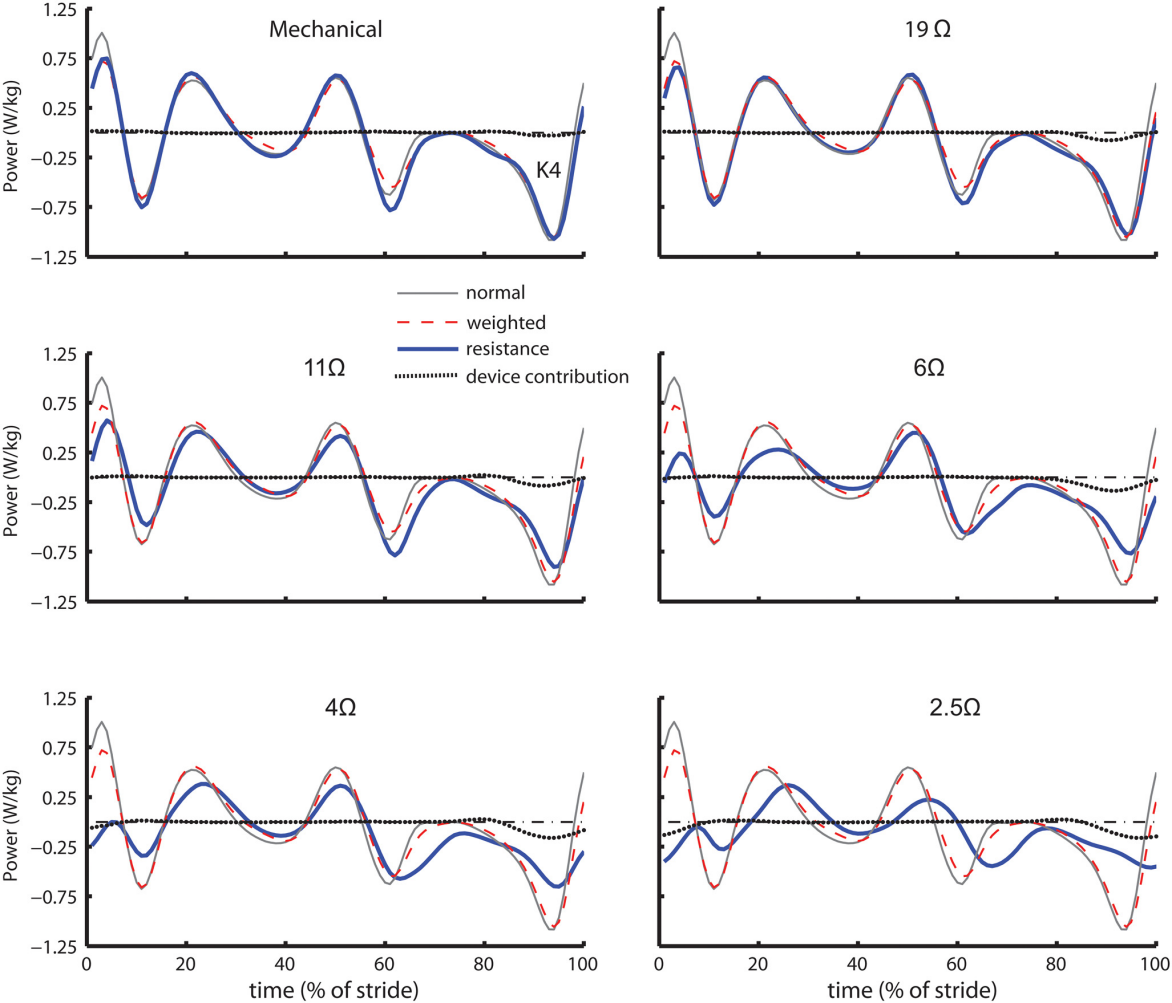
Cable contribution on the knee joint power. The mean cable power contribution (dotted) on the knee for mechanical engagement and all electrical engagement trials (19Ω, 11Ω, 6Ω, 4Ω, and 2.5Ω) in comparison with the mean knee power for normal walking condition (thin dashed line), weighted walking condition (thin solid line) and the electrical resistance trial (thick solid line).

The lower limb-driven energy harvester had a proper actuation timing, because the device provided negative work assistance to the knee flexor muscles at the K4 region of the gait cycle (Fig 7) and did not affect other regions. By comparing the areas of the knee joint power and the device power, both in the K4 region, we determined the device contribution to the user’s knee power. We found that the device contributed 7% of the total negative work during the mechanical engagement condition. This contribution increased from 10% to 24% as the electrical resistance decreased from 19Ω and 4Ω respectively. Gait adaption to the higher load applied by the device during the lowest electrical resistance (2.5Ω) most likely led to the slight decrease in device contribution (21%) relative to the 4Ω condition. The savings achieved from the negative work assistance at lower resistances (19Ω to 6Ω) might have overcome the metabolic penalty induced from alternations in walking kinematics such as step length/frequency and COM movement, resulting in a smaller or negative COH (Fig 5A). As the electrical resistance decreased to 4Ω and 2.5Ω, the negative knee power region K4 was shifted into the start of the double support phase (Fig 7). Consequently, the benefits gained from assisting negative muscle work in the K4 region could not offset the extra positive muscle work required in the other regions and the metabolic cost increased associated with kinematic alterations.

**Fig 8:**
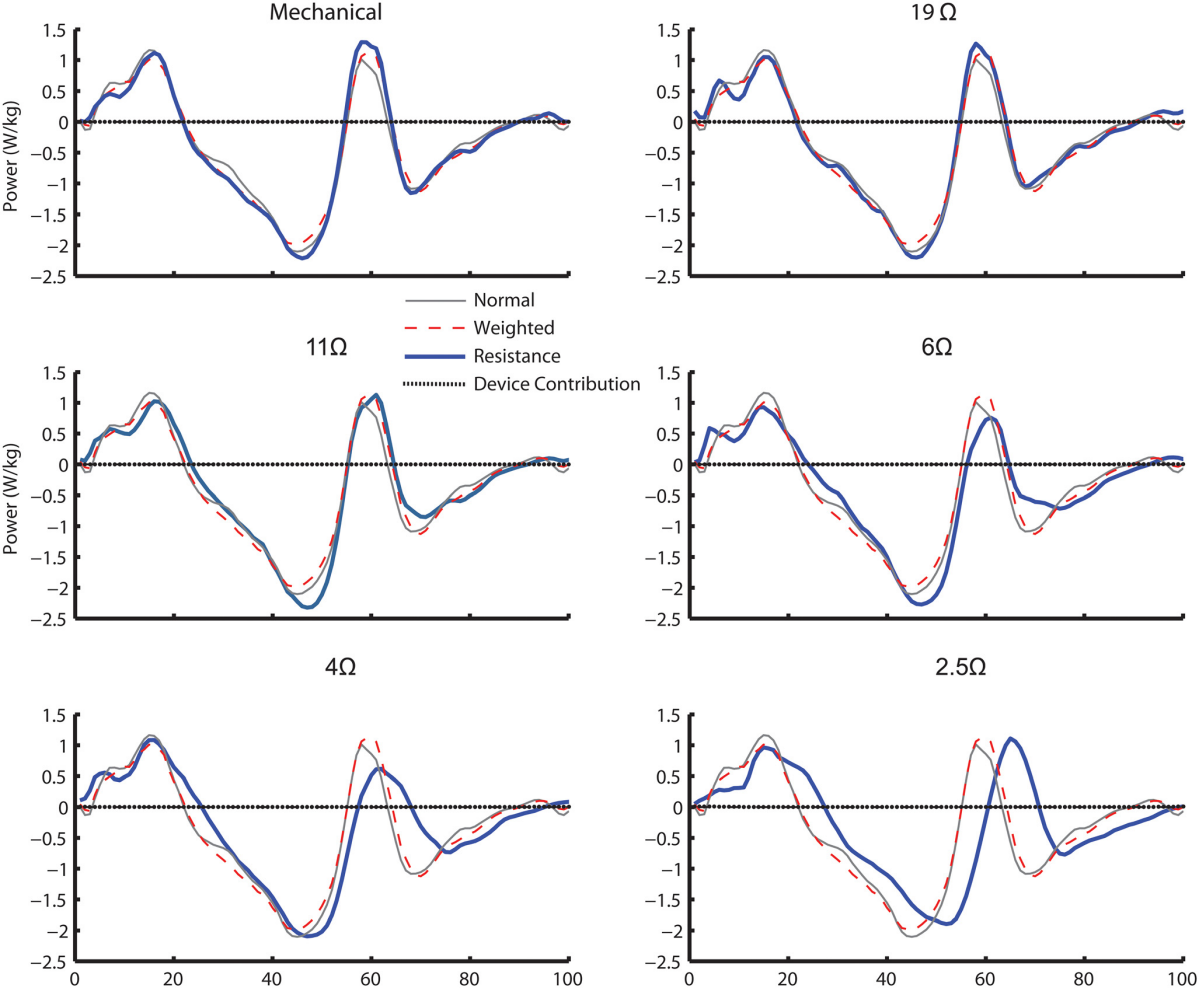
Cable contribution on the hip joint power. The mean cable power contribution (dotted) on the hip for mechanical engagement and all electrical engagement trials (19Ω, 11Ω, 6Ω, 4Ω, and 2.5Ω) in comparison with the mean hip power for normal walking condition (thin dashed line), weighted walking condition (thin solid line) and the electrical resistance condition (thick solid line).

The device did not directly contribute to the hip joint power (Fig 8). This is because the moment arm about the hip was under 0.08m during the period of maximum cable force (approximately 35N). The shift observed in hip power (4Ω and 2.5Ω) was most likely due to the compensation for changes in knee kinematics.

The results indicated that the electrical resistance of 6Ω is the optimal load for the harvester with the smallest TCOH of 4.0. With a device efficiency of 70%, the harvester produced 5.2W of electricity. Under this condition, the lower limb-driven energy harvester required the least amount of user effort in generating 5W of electricity when comparing with other existing harvesters [2, 3] and the conventional power generation method.

## Conclusions

Besides the advantage of harvesting energy during daily activities, the findings demonstrated that the lower limb-driven energy harvester is an energetically efficient alternative to the conventional power generation method in producing electricity. The results of the current study indicate that the electricity produced was mostly generated from negative knee joint work, and was associated with a small additional effort from the user. This study also identified two key factors that affected the harvester performance: the device carrying cost and the cost associated with alterations in walking mechanics. Future research on energy harvester should focus on producing useful amounts of electricity, without dramatically increasing the device carrying cost or adversely altering the user’s walking mechanics.

## Supporting Information

S1 Text Supporting information summary. (DOCX)

S1 Dataset Dataset of metabolic power, electrical power and mechanical power. (XLSX)

S2 Dataset Dataset of kinematics and kinetics. (ZIP)

S3 Dataset MATLAB program for plotting kinematic and kinetic data. (ZIP)
