# Supplementary material for: Generating Electricity during Walking with a Lower Limb-Driven Energy Harvester: Targeting a Minimum User Effort
Source: PLoS One. 2015 Jun 3;10(6):e0127635. doi: 10.1371/journal.pone.0127635 (PMC4454656; doi:10.1371/journal.pone.0127635)
Supplement: S1 Text — (DOCX) [file pone.0127635.s001.docx]

# Supporting Information Summary

This document gives a brief outline of the provided data for the paper entitled: Generating Electricity during Walking with a Lower Limb-driven Energy Harvester:  Targeting a Minimum User Effort.

. The data files include:

Table 1: The file name, size and information provided.

| File Name | File Size | Information |
| --- | --- | --- |
| Results_PLOSONE.xlsx | 7.93 KB | Metabolic power, electrical and mechanical power |
| Plotting_PLOSONE.m | 1.31 KB | Plotting data file |
| EH3_Data_PLOSONE.mat | 49.9 MB | Joint angles, torque, and power |
|  |  | Cable torque and power on the hip and knee |
|  |  | COM location |

# Excel File (“EH3_Data_PLOSONE.xlsx”)

This spreadsheet contains a table for the ten subjects (S1-S10). Within each table, the average metabolic power [W] that was determined over the 3^rd^ quarter of each ten minute trial is provided. Each table also includes the average mechanical and electrical power [W] over eight consecutive steps for each of the mechanical and electrical trials. The device efficiency [%] is calculated from this mechanical and electrical power (Elec. Pwr. / Mech. Pwr.). The cost of harvest (COH) and total cost of harvest (TCOH) for each electrical trial is provided.

# .mat File (“EHE_Data_PLOSONE”)

The file is divided into multiple levels of data. First, the data is separated by subject (1:10). Then, for each subject there are eight different trials (1:8). These trails include:

1. Normal Walking
2. Weighted Walking
3. Mechanical Engagement
4. Electrical Engagement 19 Ohms
5. Electrical Engagement 11 Ohms
6. Electrical Engagement 6 Ohms
7. Electrical Engagement 4 Ohms
8. Electrical Engagement 2.5 Ohms

Each trial contains the following information:

1. Joint angles, torque and power for the ankle, knee, and hip
2. The cable’s torque and power for the knee and hip.
3. Center of mass location
4. Step frequency, time, and length

1-3 are provided for the eight consecutive segment steps, eight normalized steps, as well as the mean and standard deviation for those eight steps. 4 is provided for each segmented step (eight in total), as well as the mean and standard deviation of those eight steps. The data structure is shown in Fig. 1.

Figure 1. Data structure of the kinematic and kinetic data
